# Cardiac arrhythmias in STEMI patients in ICU: study on occurrence in first 48 h and correlation with age, sex, infarction site, and risk factors

**DOI:** 10.1097/MS9.0000000000001264

**Published:** 2023-09-05

**Authors:** Mahmoud Alkatib, Abdul Rahman Naeem Alkotyfan, Mohammed Moutaz Alshaghel, Marwan Shamiyeh

**Affiliations:** a Faculty of Medicine, Syrian Private University, Damascus, Syria; b Faculty of Medicine, Department of Internal Medicine, Syrian Private University, Damascus, Syria; c Faculty of Medicine, Aleppo University, Aleppo, Syria

**Keywords:** arrhythmias, ICU, infarction, myocardial, STEMI

## Abstract

**Introduction:**

Acute myocardial infarction (AMI) is one of the leading causes of death in the developed world. The spread of the disease approaches three million people worldwide, with more than one million deaths in the United States annually. Myocardial ischemia and infarction can lead to electrophysiological and metabolic alterations that result in potentially fatal arrhythmias, some of which may be asymptomatic. About 90% of patients with AMI develop some form of arrhythmia during or immediately after the event, and in 25% of patients, these arrhythmias appear within the first 48 h. The most common cause of death in patients with AMI in pre-hospitalization is ventricular tachycardia/ventricular fibrillation (VT/VF).

**Methods:**

A cross-sectional study targeting 150 patients with myocardial infarction attending tertiary hospital. According to certain acceptance and exclusion criteria.

**Results:**

The sample consisted of 150 patients who suffered from heart infarction, the mean age of patients in the sample was 59.41 years with a standard deviation of 11.02 years and range of 28–90. Males constituted the largest portion of patients, with 112 males, that is 75%. The study identified that the anterior wall was the most frequent location for myocardial infarction among patients, with 64% of patients experiencing an infarction in this area. Additionally, ventricular fibrillation was the most commonly occurring arrhythmia, affecting 27% of myocardial infarction patients in the study.

**Recommendations:**

One of the most important recommendations of our study is the necessity of keeping the patient under observation for at least 48 h after myocardial infarction within the hospital to monitor the ECG (Holter) in order to detect arrhythmias. Detection of arrhythmias in every patient with extensive anterior, lateral, or posterior myocardial infarction. And the need to know and take into account ventricular fibrillation and how to manage it in every patient with a heart infarction. And conducting future studies, including a larger number of patients, to study cardiac arrhythmias more precisely.

## Introduction

Highlights

- The prevalence of myocardial infarction is high, with millions of people worldwide affected and over a million deaths in the United States annually. Arrhythmias are a common occurrence in patients with myocardial infarction, with up to 30% of patients developing them, most commonly within the first 24–48 h of the infarction.
- Sudden cardiac death (SCD) is often associated with arrhythmias and is a leading cause of death in patients with pre-hospital acute myocardial infarction (AMI), with ventricular tachycardia/ventricular fibrillation (VT/VF) being the most common cause of death. Different factors play a role in the development of arrhythmias in these patients, including hypoxia, changes in ionic current, an imbalance of electrolytes, increased automatism of the Purkinje system, and increased efferent sympathetic activity.
- There are several types of arrhythmias that may occur in the course of acute coronary syndrome (ACS), including ventricular arrhythmias and supraventricular arrhythmias. Ventricular arrhythmias often occur early after the onset of AMI and are associated with a genetic predisposition. The current from the ischemic zone is an important arrhythmia mechanism.

Arrhythmias are abnormal disturbances in the normal electrical rhythm of the heart, whether in the origination, propagation, or velocity of the impulse. They are divided into three types including extrasystole, rapid arrhythmias, and slow arrhythmias. About 30% of patients with myocardial infarction often develop arrhythmias, most of which occur within the first 24–48 h of the infarction^1^. Acute myocardial infarction (AMI) is one of the leading causes of death in the developed world. The prevalence of the disease approaches three million people worldwide, with more than a million deaths in the United States annually. Myocardial infarction results in irreversible damage to the heart muscle due to a lack of oxygen. It may impair diastolic and systolic function and make the patient susceptible to arrhythmias.

In addition, myocardial infarction can lead to a number of serious complications. The primary step in treatment is cardiac reperfusion and restoration of blood flow. The earlier treatment (within 6 h of onset of symptoms), the better the prognosis^2^.

Ischemia and myocardial infarction can cause electrophysiological and metabolic changes that result in asymptomatic or even potentially life-threatening arrhythmias. About 75% of patients with AMI develop arrhythmias during the peri-infarction period. The CARISMA trial reported the incidence of arrhythmias in AMI as 28% new-onset atrial fibrillation, 13% non-persistent ventricular tachycardia, 10% high-grade atrioventricular block (30 bpm for 8 s, 7% sinus bradycardia (30 bpm for 8 s), 5% sinus cardiac arrest (5 s), 3% persistent ventricular tachycardia, and 3% ventricular fibrillation. Sudden cardiac death (SCD) is often associated with arrhythmias, and about half of all deaths occur before patients reach the hospital. The most common cause of death in a patient with pre-hospital AMI is ventricular tachycardia/ventricular fibrillation (VT/VF)^3,4^. In ischemia, different factors play a role in arrhythmias. Hypoxia, changes in the ionic current, and an imbalance of electrolytes cause the development of arrhythmias. In addition, the increased automatism of the Purkinje systems and of the myocardium results from involuntary cardiac dysfunction. It increases efferent sympathetic activity, catecholamine concentration, and local catecholamine release from nerve endings in the same myocardium. Furthermore, whole-wall infarction can lead to disruption of the afferent and efferent sympathetic nerves that innervate parts of the heart muscle distal from the area of ​​the infarct. This involuntary imbalance leads to the development of an arrhythmia. Other mechanisms include increased levels of free fatty acids and oxygen-derived free radicals, which may also play a role in the development of arrhythmias in acute coronary syndrome (ACS). There are several types of arrhythmias that may occur in the course of ACS, such as ventricular arrhythmias (ventricular tachycardia, ventricular fibrillation) and supraventricular (sinus arrhythmias, paroxysmal supraventricular arrhythmias, atrial fibrillation, tachycardia, and focal tachycardia). Ventricular arrhythmias often occur early after the onset of AMI. It presents as polymorphic VT or VF in a small proportion of patients with acute ischemia and is often associated with a genetic predisposition. Ventricular tachycardia is more common with increasing duration of ischemia, but as myocardial involvement spreads, the incidence of ventricular tachycardia decreases. An important arrhythmia mechanism is the current from the ischemic/ischemic zone and its transmission to the non-ischemic zone^4,5^.

## Methods

### Study design

A retrospective cross-sectional study was conducted in the Department of Cardiac Intensive Care Unit of a tertiary hospital in Damascus city, over 3 years from January 2019 to June 2022.

### Inclusion and exclusion criteria

The study included all STEMI patients admitted to the cardiac intensive care between 2019 and 2022 and observed in cardiac intensive care within the first 48 h of myocardial infarction. Patients with NSTEMI infarction, angina pectoris, patients who underwent PCI during the first 48 h of infarction, patients who were not fully monitored during the first 48 h, and patients for whom there was a lack of information required for the study were excluded.

### Data collection

The data related to this study were collected using paper questionnaires. The questionnaire was designed based on previous studies, in addition to the information available in the medical files, and was supervised by the professor in charge of the study. The questionnaire included two parts: the first was about sample demographics in addition to information about lifestyle, and the second part discussed the location of the myocardial infarction and the arrhythmias that occurred.

### Ethical considerations

The study received ethical approval from the Institutional Review Board (IRB) of the Faculty of Medicine at the University.

### Statistical analysis

The data from the paper questionnaires were entered into a Google Form and then exported as an Excel file in order to clean the data in preparation for statistical analysis. SPSS version 25 was used to analyze the data. Descriptive analysis was conducted, including frequencies and percentages for all variables in the study, and inferential analysis included studying the existence of relationships and correlations between many of the variables studied using Chi-square.

The work has been reported in line with the STROCSS criteria^6^.

## Results

### Socio-economic variables and history

The sample consisted of 150 patients who suffered from myocardial infarction. The average age of the patients in the sample was 59.41 years with a standard deviation of 11.02 years. The oldest patient in the sample was 90 years old, while the youngest was 28 years old. Males constituted the largest proportion of the sample, with 112 male patients, representing 75% of the total.

Out of the 150 patients in the sample, 110 patients were smokers, which represents 73% of the total sample. Additionally, 30 patients, or 20%, reported consuming alcohol, with regard to weight; only one patient had a normal weight, while 54 patients were overweight (36%), 56 patients (37%) suffered from obesity, and 39 patients (26%) had morbid obesity.

A total of 103 patients, or 69%, reported suffering from stress and anxiety. Furthermore, 64 patients, representing 43% of the sample, reported having diabetes. As for arterial tension, 72 patients, or 48%, reported suffering from high blood pressure. Finally, a total of 63 patients, or 42%, reported suffering from other diseases (Table 1).

**Table 1:** Socio-demographic and lifestyle information.

Variable	*N* (%)
Age	
Mean (SD)	59.41 ± 11.03
Sex	
Male	112 (74.7%)
Female	38 (25.3%)
Smokers	110 (73.3%)
Alcohol consumers	30 (20.0%)
Obesity	
Normal	1 (0.7%)
Overweight	54 (36.0%)
Obese	56 (37.3%)
Very obese	39 (26.0%)
Stressful lifestyle	103 (68.7%)
Diabetes mellitus 2	
HTN	72 (48.0%)
DM	64 (42.7%)
Other diseases	63 (42.0%)

### The location of the infarction and the arrhythmias that accompany it

The study found that the most common location for myocardial infarction in patients was the anterior wall of the heart, with 96 patients (64%) experiencing an infarction in this area. The lower wall of the heart was the second most common location, with 33% of patients experiencing an infarction in this area. The heart rhythm disturbances accompanying myocardial infarction varied among the patients. The most common arrhythmia was ventricular fibrillation, which was found in 40 patients (27%) who suffered from myocardial infarction. Atrial fibrillation came in the second place in terms of prevalence, with a rate of 20%. The third most common conductive disorder accompanying infarction in the sample was right bundle branch block, which was found in 24 patients (16%). Ventricular extrasystole was more common than atrial extrasystole, as it was found in 14 patients (9%), while atrial extrasystole was found in only 3 patients (2%) (Table 2).

**Table 2:** Location of MI and type of arrhythmia frequencies and percentages.

Variable	*N* (%)
Location of myocardial infraction	
Anterior	96 (64.0%)
Lateral	3 (2.0%)
Inferior	50 (33.3%)
Apical septal	1 (0.7%)
Type of arrhythmia	
Atrial fibrillation	30 (20.0%)
Ventricular fibrillation	40 (26.7%)
Sinus bradycardia	2 (1.3%)
Sinus tachycardia	5 (3.3%)
Ventricular tachycardia	12 (8.0%)
Atrial tachycardia	2 (1.3%)
Atrioventricular block 1	3 (2.0%)
Atrioventricular block 2	4 (2.7%)
Atrioventricular block 3	4 (2.7%)
Atrial extrasystole	3 (2.0%)
Ventricular extrasystole	14 (9.3%)
Right branch block	24 (16.0%)
Left branch block	16 (10.7%)
Anterior left branch block	2 (1.3%)

### The relationship between sex and the type of arrhythmia that follows infarction

By studying the relationship between the type of arrhythmia accompanying a heart infarction and the sex of the patient, we found a statistically significant relationship between the sex of the patient and the appearance of atrial tachycardia after myocardial infarction; 5% of the female patients experienced this arrhythmia, in contrast, none of the male patients were affected. The *P* value was 0.0145, indicating a meaningful relationship between these two variables (Table 3).

**Table 3:** Association between type of arrhythmia and many factors related to demographics and lifestyle.

	Sex		Smoking		Alcohol		Stress		HTN		DM2	
Type of arrhythmia	Males	Females	Non-smokers	smokers	No	Yes	No	Yes	No	Yes	No	Yes
	0.851		0.356		0.610		0.538		0.072		**0.017**	
Atrial fibrillation	22	8	10	20	23	7	8	22	20	10	23	7
	0.428		0.330		0.644		0.160		**0.012**		0.273	
Ventricular fibrillation	28	12	13	27	33	7	9	31	14	26	20	20
	0.407		0.391		0.477		0.336		0.955		0.099	
Sinus bradycardia	2	0	0	2	2	0	0	2	1	1	0	2
	0.443		0.732		0.255		0.671		0.716		0.902	
Sinus tachycardia	3	2	1	4	5	0	2	3	3	2	3	2
	0.506		0.892		0.652		0.146		0.177		0.942	
Ventricular tachycardia	8	4	3	9	9	3	6	6	4	8	7	5
	**0.015**		0.453		0.477		0.336		0.138		0.099	
Atrial tachycardia	0	2	1	1	2	0	0	2	0	2	0	2
	0.096		0.114		0.382		0.940		0.608		0.396	
Atrioventricular block 1	1	2	2	1	3	0	1	2	2	1	1	2
	0.238		0.222		0.128		0.415		0.051		0.764	
Atrioventricular block 2	4	0	0	4	2	2	2	2	4	0	2	2
	0.238		0.939		0.311		0.782		0.351		0.764	
Atrioventricular block 3	4	0	1	3	4	0	1	3	3	1	2	2
	0.308		0.291		0.560		0.237		0.531		0.741	
Atrial extrasystole	3	0	0	3	2	0	0	3	1	2	2	1
	0.724		0.271		0.123		0.401		0.686		0.263	
Ventricular extrasystole	11	3	2	12	9	5	3	11	8	6	10	4
	0.287		0.481		0.911		**0.031**		0.498		0.732	
Right branch block	20	4	5	19	19	5	12	12	14	10	13	11
	0.522		0.873		**0.034**		0.089		0.865		0.245	
Left branch block	13	3	4	12	16	0	8	8	8	8	7	9
	0.407		0.391		0.477		0.567		0.171		0.099	
Anterior left branch block	2	0	0	2	2	0	1	1	2	0	0	2

### The relationship between smoking and the type of arrhythmia that follows infarction

When we studied the relationship between smoking and the type of arrhythmia that occurs in the patient after myocardial infarction, we did not find a relationship between any of the types of arrhythmias and smoking (Table 3).

### The relationship between alcohol consumption and the type of arrhythmia that follows infarction

By studying the relationship between alcohol consumption and the type of arrhythmia associated with patients after their infarction, we found a relationship between left branch block and alcohol consumption in patients. The *P* value was 0.034, indicating a significant relationship between these two variables (Table 3).

### The relationship between stress and the type of arrhythmia that follows infarction

When we studied the presence of tension or anxiety in the patient and its relationship to the type of arrhythmia that occurred after a heart infarction, we found a statistically significant relationship between the presence of anxiety or tension in patients and the presence of right branch block as one of the arrhythmias that occurred after the infarction. The *P* value was 0.031, indicating a relationship between these two variables (Table 3).

### The relationship between diabetes and the type of arrhythmia in patients

By studying the relationship between the presence of diabetes mellitus and the type of arrhythmia associated with patients after their infarction, we found a relationship between atrial fibrillation and diabetes mellitus in patients. The *P* value was 0.016, indicating a significant relationship between these two variables (Table 3).

### The relationship between hypertension and the type of arrhythmias that follow the infarction

Regarding the relationship between the presence of high arterial tension and the type of arrhythmia occurring in patients, we found a relationship between ventricular fibrillation and the presence of high arterial tension in patients; 36% of patients suffering from high arterial tension had ventricular fibrillation, while 18% of patients without arterial tension had ventricular fibrillation (Table 3).

### The relationship between BMI and the type of arrhythmias that follow the infarction

Association between type of arrhythmia and BMI in the study population. The results suggest that there is no significant association between BMI and the occurrence of most types of arrhythmias, including atrial fibrillation, ventricular fibrillation, sinus bradycardia, sinus tachycardia, ventricular tachycardia, atrial tachycardia, atrioventricular block 1, atrioventricular block 2, atrioventricular block 3, atrial extrasystole, and ventricular extrasystole. However, there is a significant association between BMI and the occurrence of right branch block, with a higher prevalence of right branch block in overweight and obese patients compared to normal weight patients with a *P* value of 0.03 (Table 4).

**Table 4:** Association between type of arrhythmia and BMI.

Variable	Normal	Overweight	Obese 1	Obese 2	*P*
Atrial fibrillation	0	13	13	4	0.323
Ventricular fibrillation	0	15	10	15	0.145
Sinus bradycardia	0	2	0	0	0.308
Sinus tachycardia	0	1	2	2	0.849
Ventricular tachycardia	0	2	6	4	0.518
Atrial tachycardia	0	0	1	1	0.734
Atrioventricular block 1	0	1	0	2	0.374
Atrioventricular block 2	0	3	1	0	0.392
Atrioventricular block 3	0	0	3	1	0.381
Atrial extrasystole	0	0	3	0	0.162
Ventricular extrasystole	0	5	8	1	0.279
Right branch block	1	12	8	3	**0.030**
Left branch block	0	3	6	7	0.287
Anterior left branch block	0	2	0	0	0.308

### The relationship between infarction and arrhythmias

There is a significant association between the location of myocardial infarction and atrial fibrillation, ventricular fibrillation, sinus tachycardia, and atrioventricular block 3, with a *P* value of less than 0.001, 0.007, 0.016, and 0.042, respectively (Table 5).

**Table 5:** Association between type of arrhythmia and location of MI.

Variable	Anterior	Inferior	Lateral	Apical septal	*P*
Atrial fibrillation	10	18	2	0	**0.000**
Ventricular fibrillation	33	6	0	1	**0.007**
Sinus bradycardia	0	2	0	0	0.256
Sinus tachycardia	4	0	1	1	**0.016**
Ventricular tachycardia	8	4	0	0	0.948
Atrial tachycardia	2	0	0	0	0.767
Atrioventricular block 1	2	1	0	0	0.994
Atrioventricular block 2	2	2	0	0	0.901
Atrioventricular block 3	0	4	0	0	**0.042**
Atrial extrasystole	2	1	0	0	0.994
Ventricular extrasystole	8	5	1	0	0.515
Right branch block	18	6	0	0	0.594
Left branch block	11	5	0	0	0.905
Anterior left branch block	2	0	0	0	0.767

## Discussion

About 90% of patients suffering from AMI have a form of cardiac arrhythmia during or immediately after the event, and in 25% of patients, these arrhythmias may manifest during the first 48 h. Ventricular tachycardia of multiple forms or ventricular fibrillation may manifest in a minority of patients with acute infarction, including during the early phase of ACS and may have a genetic component^7^. Most AMI deaths occur due to arrhythmias including atrioventricular block, bradycardia, and supraventricular tachycardia^8,9^. This could be caused by electrolyte disturbances, which are common in the first 24 h after AMIs, such as sodium and potassium as these electrolytes play an important role in arrhythmias^10^. Although the first 24–48 h should be observed carefully, little care is given in the recovery period of AMI; however, patients are still at risk for acute arrhythmias as well as sudden death^11^.

Out of the sample, 112 males had an arrhythmia, which represents 75% of the sample, and this coincides with the global proportions that showed that males have a 1.5 times higher risk of having cardiac arrhythmias compared to females^12^. Another study revealed that males that suffer from coronary artery disease have a 5.4 times higher risk of atrial fibrillation than females^13^. The mean age of patients in the sample was 59 years old, which is similar to the global mean age of patients with arrhythmias after myocardial infarction, which is 56.6 years old, as ages ranged between 25 and 89 years^10^. The cause of the high mean age of patients is the increased risk of myocardial infarction with age, as age is one of the independent risk factors of developing myocardial infarction; in addition to that, age is considered a risk factor for arrhythmias, and age is positively correlated with an increased risk of cardiac diseases including arrhythmias. The severity of arrhythmias may also increase with age^14^.

The number of myocardial infarction patients who smoke and developed an arrhythmia is 110 patients making 73% of the sample, as it is likely that other components such as carbon monoxide and the oxidative overload may cause arrhythmias. Lastly, smoking cigarettes could cause coronary artery disease and chronic obstructive pulmonary disease, which may in turn lead to arrhythmias^15^. Fifty-six patients from the sample suffered from obesity when diagnosed with infarction and arrhythmias, which was confirmed by global statistical studies that revealed that patients who suffered from severe obesity have almost double the risk of SCD compared to peers of the same age^16,17^ since SCD occurs due to arrhythmias.

Atrial tachycardia was seen in five patients with myocardial infarction, as global studies have revealed that prolonged arrhythmias after infarction are associated with an increased risk of death. Studies also revealed that the presence or development of atrial tachycardia within the first 3 days after myocardial infarction has increased risk of death^18^. Right and left bundle branch blocks were observed in 14 and 28 patients, respectively, in our study, which matches the results of global studies that revealed the incidence of left bundle branch block is 3.4%, and the incidence of right bundle branch block is 4.3%, as patients who suffered from left branch blocks, when these patients were monitored, they had worse outcomes and an increased death rate^19^.

Hypertension is usually related to arrhythmias in patients who suffer from cardiovascular diseases. Our results revealed a correlation between patients who have hypertension have a higher tendency to get atrial fibrillation after infarction occurs, with a *P* value of 0.0120, and this has been supported by several clinical trials and case reports that suggest a possible correlation between hypertension and atrial and ventricular tachycardia despite unclear physiological etiology^20^.

Our study revealed a relationship of statistical relevance between diabetes and atrial fibrillation and myocardial infarction, which agrees with most global studies that have shown that diabetes plays an important role in many types of arrhythmias, and the association between diabetes and atrial fibrillation has been extensively studied^21^. A 38-year follow-up of patients identified diabetes as an independent factor for atrial fibrillation^12^.

## Conclusions

Our results suggest several conclusions that should be taken into consideration and these include: the necessity of monitoring patients for arrhythmias within the hospital for 48 h after infarction occurs, using a Holter monitor. Arrhythmias should also be evaluated in patients with anterior, posterior, or lateral infarctions. It is also crucial to consider atrial fibrillation and its management in every patient with infarction. Future studies should include more hospitals and patients to further investigate arrhythmias. Patients should be educated about the negative effects of obesity on heart health and the importance of maintaining a healthy weight.

## Ethical approval

The study received ethical approval from the Institutional Review Board (IRB) of the Faculty of Medicine at Syrian Private University, under registration number 4520 for the year 2022. Additionally, it was also authorized by the administration of Damascus Hospital.

## Consent

Written informed consent was obtained from the patient for publication and any accompanying images. A copy of the written consent is available for review by the Editor-in-Chief of this journal on request.

For Minors: Written informed consent was obtained from the patient’s parents/legal guardian for publication and any accompanying images. A copy of the written consent is available for review by the Editor-in-Chief of this journal on request.

## Sources of funding

This research received no specific grant from SPU or any other funding agency in the public, commercial, or non-profit sectors.

## Author contribution

M,S. assisted with the research idea and supervised the study. M.A. and A.R.N.A. created the online questionnaire, collected the data, and wrote the manuscript. M.M.A. did the statistical analysis, proofread, and formatted the final manuscript.

## Conflicts of interest disclosure

None of the authors have any conflicts of interest. The authors alone are responsible for the content and writing of the article. No conflicts of interest are declared.

## Research registration unique identifying number (UIN)

1. Name of the registry: Research Registry.
2. Unique identifying number or registration ID: researchregistry9083.
3. Hyperlink to your specific registration (must be publicly accessible and will be checked): https://www.researchregistry.com/browse-theregistry#home/registrationdetails/64726bdfebacf7002838f3b0/

## Guarantor

Mahmoud Alkatib.

## Data availability statement

All data related to this paper’s conclusion are available and stored by the authors. All data are available from the corresponding author on reasonable request.

## Provenance and peer review

Not invited.
